# Recent Advances in Aptamer-Based Biosensors for Bacterial Detection

**DOI:** 10.3390/bios14050210

**Published:** 2024-04-23

**Authors:** Vincent Léguillier, Brahim Heddi, Jasmina Vidic

**Affiliations:** 1 INRAE, AgroParisTech, Micalis Institut, Université Paris-Saclay, UMR 1319, 78350 Jouy-en-Josas, France; vincent.leguillier@inrae.fr; 2 ENS Paris-Saclay, Laboratoire de Biologie et Pharmacologie Appliquée (LBPA), UMR8113 CNRS, 91190 Gif-sur-Yvette, France

**Keywords:** detection, biosensors, aptamers, bacterial pathogens, food security

## Abstract

The rapid and sensitive detection of pathogenic bacteria is becoming increasingly important for the timely prevention of contamination and the treatment of infections. Biosensors based on nucleic acid aptamers, integrated with optical, electrochemical, and mass-sensitive analytical techniques, have garnered intense interest because of their versatility, cost-efficiency, and ability to exhibit high affinity and specificity in binding bacterial biomarkers, toxins, and whole cells. This review highlights the development of aptamers, their structural characterization, and the chemical modifications enabling optimized recognition properties and enhanced stability in complex biological matrices. Furthermore, recent examples of aptasensors for the detection of bacterial cells, biomarkers, and toxins are discussed. Finally, we explore the barriers to and discuss perspectives on the application of aptamer-based bacterial detection.

## 1. Introduction

Pathogenic microorganisms have posed a serious challenge to human health and wellbeing throughout history. They encompass a wide range of bacteria, viruses, fungi, and parasites that can be transmitted through food, water, soil, and air or through contact with infected individuals or animals. A survey for pathogens is one of the most important elements in tracing infectious diseases, determining the distribution of agents according to sites, and preventing their spread. Among bacterial pathogens, of particular concern are those resistant to the antibacterial drugs commonly used to treat infections, such as pathogenic strains designated by the acronym ESKAPE (*Enterococcus faecium*, *Staphylococcus aureus*, *Klebsiella pneumoniae*, *Acinetobacter baumannii*, *Pseudomonas aeruginosa*, and *Enterobacter* species). These bacteria cause the majority of nosocomial and community-acquired infections worldwide [1,2]. In addition, more than 200 identified bacterial pathogens are associated with foodborne illnesses, which can occur through either foodborne infection (when ingested bacteria colonize and multiply in the human host) or foodborne intoxication (resulting from the consumption of food containing bacterial toxins). The most widespread foodborne and waterborne bacteria are *Escherichia coli* O157:H7, *Salmonella* spp., *Clostridium perfringens*, *Cronobacter sakazakii*, *Bacillus cereus*, *Campylobacter jejuni*, *S. aureus*, *Listeria monocytogenes*, *Shigella* spp., *Clostridium botulinum*, *Vibrio* spp., and *Yersinia enterocoli* [3,4]. Some foodborne bacteria, such as *L. monocytogenes*, pose a significant risk due to their high mortality rates, while others, such as *Salmonella* and *C. jejuni*, present a high frequency of occurrence. Moreover, some pathogenic bacteria are spore-forming (e.g., *Clostridium botulinum*, *Bacillus cereus*, *Clostridium perfringens*, *Clostridioides difficile*) [5,6,7]. In their sporulated forms, bacteria are highly heat-resistant and survive most disinfection procedures used in the food industry or hospitals. Some bacterial toxins, such as staphylococcal enterotoxin B (SEB) and cereulide, are resistant to heat and may persist in food products for long periods. Additionally, bacteria can form biofilms, which provide them with further protection against disinfection measures, making eradication more challenging.

Bacterial detection holds practical importance in the pharmaceutical industry, environmental control, and food safety. Foodborne infections and intoxication resulting from contaminated beverages or food are considered serious problems and have become more prevalent recently. In 2015, the World Health Organization (WHO) estimated that there had been 600 million cases of foodborne illnesses, representing 11.6% of the world’s population and resulting in no fewer than 420,000 deaths. In 2019, it was estimated that 7.7 million deaths had been caused by pathogenic bacteria [8]. In the interest of public health and consumer protection, it is evident that there is a need to concentrate efforts to develop new methods for production control, including new detection techniques.

To reduce the risks and control disease progression, a diverse range of methods have been developed for pathogen detection, including culture methods and molecular methods, such as those based on nucleic acid amplification (e.g., polymerase chain reaction (PCR)) [9,10,11] and the enzyme-linked immunosorbent assay (ELISA) [12]. Although they are the most frequently used methods for pathogen detection, they show many disadvantages that prevent efficient health security and sanitary control. Methods based on cultivation are time-consuming as they require several steps for bacterial enrichment, isolation, and identification. In addition, they can be performed only by qualified personnel and within a laboratory setting. Molecular methods are less time-consuming, but they also require a pre-enrichment step to increase the number of targeted bacterial cells and to prevent the detection of dead bacteria. In an effort to address these shortcomings, in recent years, the rapid development of biosensors for bacterial detection has been pursued. Biosensors are analytical devices that integrate a bioreceptor, which assures the recognition of the target, with a transducer, which converts the recognition event into a measurable signal, enabling the detection of chemical or biological analytes [13].

**Figure 1:**
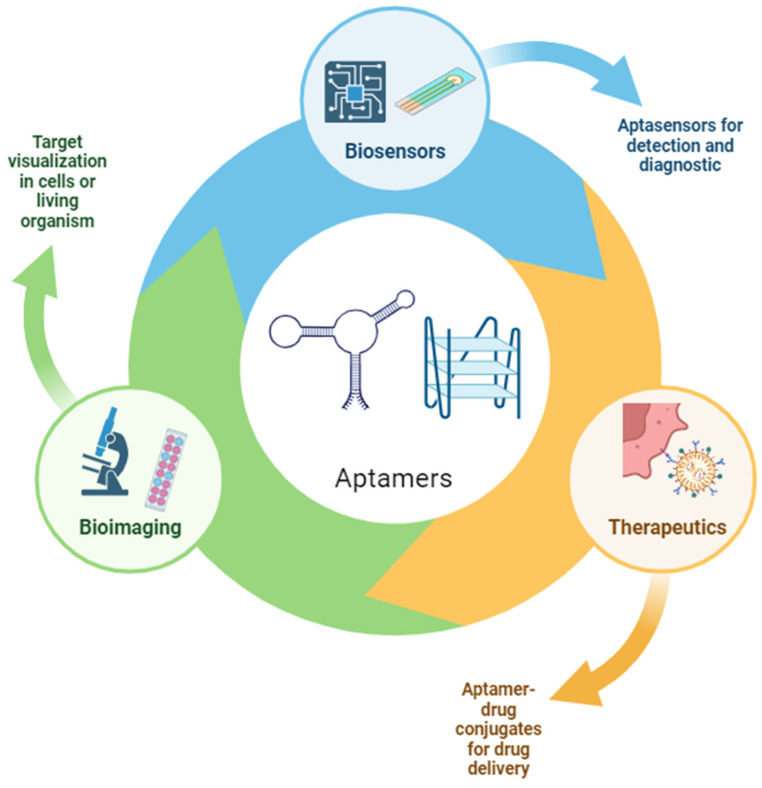
Major aptamer characteristics and applications. Figure created with BioRender.com. (accessed on 2 April 2024).

Among bioreceptors, aptamers, which are small, artificial, single-stranded DNA or RNA molecules (usually 20–100 nucleotides long), capable of binding to targets with high specificity and sensitivity, have emerged as specific sensing elements. Aptamers usually have no biological function but exhibit a strong affinity for specific targets through structural recognition, similar to the natural interactions observed between antigens and antibodies. Aptamers possess several advantages over expensive antibodies and stand out as strong candidates to replace them. They can selectively recognize the desired target with a strong affinity and specificity, and they can be easily customized to accommodate changes [14,15,16]. Due to such properties, aptamers have attracted significant attention in recent years for their potential to revolutionize various fields, including diagnostics and therapeutics, as illustrated in Figure 1.

This review focuses on the latest advancements in the use of aptamers for bacterial detection, and recent studies are discussed. In particular, we consider the strategies for the selection of aptamers and their kinetics, thermodynamics, and structural characterization. Furthermore, we discuss the principal methodologies and techniques used to enhance the overall efficiency and functionality of aptamers for improved target detection. Recently, two reviews have described aptamer identification, the transducer technologies used in aptasensors and nanomaterials for aptasensors [17,18,19,20,21,22,23]. Differing from these reports, the current review aims to demonstrate the present circumstances and the prospects of various aptasensors for the detection of pathogenic bacteria. In addition, this review analyzes barriers to the use of aptasensors and may assist researchers in developing user-friendly, low-cost, labor-saving, and point-of-care diagnostic devices.

## 2. Aptamer Selection Strategies for Bacterial Pathogen Detection

Aptamers, first developed in the early 1990s by two independent teams, Ellington and Szostak [24] and Tuerk and Gold [25], are distinct nucleic acid sequences with selective and specific binding to a target. These sequences are selected from a large pool of nucleic acids with an extensive sequence capacity, ranging between 10^13^ and 10^16^, utilizing an in vitro selection technique known as the systematic evolution of ligands by exponential enrichment (SELEX) [26,27]. SELEX is a series of selection and amplification steps in which a large pool of nucleic acid molecules binds to the desired target molecules under specific conditions, including a specific temperature and salt concentration. The molecules that bind to the target are isolated from those that remain unbound, and then they are amplified to generate a newly enriched population. This cycle is repeated until a small population of highly specific molecules is obtained.

The SELEX technique has undergone numerous modifications and improvements, making it more efficient, reliable, and affordable for the identification of aptamers, as detailed in [28,29,30,31]. The success of SELEX experiments relies heavily on the initial population. The initial pool of nucleic acids used in SELEX contains a random nucleotide region flanked by two constant regions, the primers, that enable amplification in the SELEX process to enrich the sequences that bind to the target molecule. The primers are usually nonessential nucleotides and are not involved in the folding process or used to recognize the target [32]. However, in some outlier cases, the primer region can participate in the aptamer structure’s formation, particularly for a short random sequence [33,34,35,36]. The influence of the random region length on aptamers has been studied, but overall, there is no significant correlation between the length and the aptamer’s affinity and stability [37,38]. However, a longer random region enhances the complexity of the secondary and tertiary structures, while a shorter one provides better coverage and gives production advantages [39]. The optimal random region length was estimated to be 20–50 nt, while the primers’ length was 20 nt [32]. More complex initial nucleic acid libraries are increasingly being used. For instance, secondary structures have been included in the initial random sequences to increase the likelihood of complex functional folds. A library containing modified bases, including sugar, backbone, and base modifications, has been shown to successfully generate aptamers, although several modified enzymes need to be utilized for the SELEX process [40,41,42].

Bacterial pathogen detection can be achieved indirectly by developing aptamers against secretory molecules such as toxins. Some bacterial toxins can survive pasteurization and resist high temperatures (<100 °C), indicating the bacteria’s past or current presence. Thus, the indirect detection of bacteria is important for food, medical, and environmental safety. Toxins pose significant risks to food safety and the environment, with even low concentrations potentially harming human health. The detection of these toxins relies on labor-intensive processes and expensive laboratory equipment. However, the selection of aptamers to target small-molecule toxins faces challenges such as the inefficient separation of bound and unbound DNA, limited binding motifs on target surfaces, a lack of chemical groups for target immobilization, and difficulties in distinguishing between molecules with similar structures. Among the few aptamers for toxins documented in the current literature are enterotoxins, cholera toxins, and botulinum toxins, produced by *S. aureus*, *Vibrio cholerae*, and *C. botulinum*, respectively [43,44,45,46,47,48,49,50,51,52].

Another and more direct approach employs aptamers selected to act against a specific molecule of bacteria. Bacterial cell surfaces present a multitude of molecules, particularly proteins, including surface epitopes such as membrane and cell wall proteins or peptidoglycans, which can be chosen as a target for aptamer selection. However, this strategy requires prior knowledge of the surface molecule and its production in a pure soluble form, which may not accurately represent its cellular context. Furthermore, the epitope might not be accessible in its native environment. Achieving selectivity against a specific strain of bacteria requires the surface molecule to be exclusive to this strain, which presents a considerable challenge. Consequently, this strategy has been primarily successful in detecting a type of bacterium rather than a particular strain of bacteria. For instance, RNA aptamers have been reported that target teichoic acid in *S. aureus* [53], a component of the cell wall of most Gram-positive bacteria, as well as peptidoglycan-specific aptamers for the detection of *S. aureus* and *E. coli* [54] and, more recently, aptamers targeting isdA, a crucial surface protein for *S. aureus*’ survival and colonization. Over time, this protein has gradually become a key marker for the detection of this bacterium [55,56].

Aptamers can be selected to detect a specific bacterium directly by using whole cells or spores as targets. This approach is termed cell-based aptamer selection or cell-SELEX [57,58]. It was first introduced in 1998 by Morris et al., who used human red blood cells to generate aptamers capable of selectively binding to unidentified molecular biomarkers present on the surfaces of live cells [59]. Many aptamers that selectively bind Gram-negative and Gram-positive bacteria, such as *E. coli* [60,61], *C. jejuni* [62,63], *Streptococcus pyogenes* [64], *S. aureus* [65,66,67,68], *B. cereus* [69], *Salmonella typhimurium* [70], or *Listeria* spp. [71], can be found in the literature.

Cell-SELEX was shown to be effective in generating specific aptamers against a particular bacterium or even a particular clone. Similarly, the cell-SELEX procedure can be used with bacterial spores to develop specific aptamers. In their sporulated forms, bacteria exhibit higher resistance to various stress factors, such as UV radiation and heat, than the corresponding growing cells [72,73]. The conventional microbiological methods used to detect bacterial spores have lengthier procedures than those used for vegetative cell detection. This is because the spores are first subjected to germination; then, their vegetative cells are characterized. Therefore, conventional detection methods such as PCR or immunological assays are not adapted to ensure full safety. In contrast, aptamers that bind to the surfaces of bacterial spores offer the potential for rapid and direct detection. In particular, aptamers have been developed and used in different aptasensors for the detection of spores from *Bacillus* species, which are ubiquitously present in air, water, and soil [74,75,76].

The procedure of cell-SELEX does not require purification or prior knowledge of a specific targeted surface epitope. Moreover, the proteins on the cell surface represent a more physiological and natural folding condition than purified proteins. However, cell-SELEX might be difficult to perform because the bacterial cell surface is negatively charged, leading to DNA repulsion from the cell surface [77]. Additionally, the process can introduce selection biases. For instance, the presence of dead cells in a suspension can result in non-specific binding, strongly influencing the selection. Another challenge lies in identifying the specific target(s) of the selected aptamers. The majority of the literature using cell-SELEX fails to demonstrate a comprehensive understanding of the targets.

Alternative in silico strategies have been developed in the last few decades. These methods are based on the computational screening of a large library of aptamer sequences to fit modeled target structures. These techniques require 3D structure predictions, through docking methodologies using software such as HADDOCK, AutoDock, or Xdock, enabling the study of aptamer folding and the selection of structural patterns responsible for the aptamer/target interaction based on the predicted affinity and Gibbs free energy (∆G) [78,79,80]. The in silico method has been successfully conducted to select aptamers for bacterial surface proteins [81,82,83]. Another example is a recently employed computational screening method used to select antimicrobial aptamers against the pathogen *Pseudomonas aeruginosa* [84]. The in silico strategy is a powerful and complementary tool to SELEX for the development of new aptamers against pathogenic bacteria. As a computational-based approach, it offers time savings and cost-effectiveness compared to SELEX. Nevertheless, in silico methods still require knowledge of aptamers’ tridimensional (3D) structures and in vitro testing to confirm the binding interaction between the selected aptamers and the targets.

## 3. Aptamer Characterization: Affinity, Stability, and Structure

Aptamers are typically short, single-stranded nucleic acids, either DNA or RNA molecules, that can fold into various 3D structures. Nucleic acids are chemically and structurally stable. Both share similar functionalities and have their advantages: RNA aptamers offer more diverse and complex 3D structures that can enhance the binding specificity, and DNA is known for its stability and lower production costs [15]. They can recognize and bind their targets with high specificity and strong affinity in the nano- and picomolar ranges. The structural conformation of an aptamer depends not only on its base composition but also on its environment, including the pH, salt type, and ionic force. These parameters have a significant influence on the formation and stability of specific conformations of aptamers; they are particularly important for intercalated motif (I-motif) or G-quadruplex structures [85,86,87,88].

**Figure 2:**
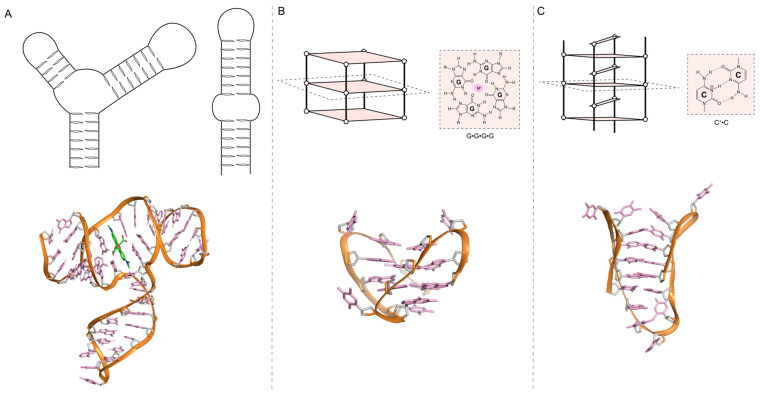
Schematic aptamer structures of three-way junctions and hairpins (**A**), G-quadruplexes and G-tetrads (**B**) and I-motifs and C•C^+^ base pairs (**C**). Below each schematic, a ribbon view of a representative structure in each category is displayed, [92] PDB code 6GzK, (**A**) [93] PDB code 148D, (**B**) [94] PDB code 1A83 and (**C**) PDB. The backbone is colored orange, and the bases are pink.

It has been shown that aptamers can adopt various tertiary structures, which can be categorized into three types: structures containing duplexes, loops, bulges (e.g., hairpins, pseudoknots, triplexes, and three-way junctions), G-quadruplexes, and I-motifs (Figure 2). The loop, bulge length, and base composition (Figure 2A), play a crucial role in the structure and function of aptamers, influencing their binding affinity and specificity [89,90,91].

Structures containing a duplex stem with different loops and bulges are very diverse (the loop length number and composition can vary), ranging from simple hairpins to pseudoknots (Figure 2A), all resulting in a spectrum of distinct conformations that participate in the target’s selection. G-quadruplexes are four-stranded non-canonical nucleic acid structures that can form under physiological conditions [95]. G-quadruplex structures are stacked G-tetrads formed by four hydrogen-bonded guanines stabilized by cations (Figure 2B). They display remarkable structural diversity, capable of adopting various conformations depending on the sequence composition, loop size, salt type, and concentration [96,97]. In terms of stability, they exhibit high thermal and nuclease resistance. Their structural features include a large hydrophobic planar surface (G-tetrad, Figure 2B) and high electrostatic density, providing a stable scaffold for aptamer selection through iterative rounds of amplification and selection. These unique properties contribute to their widespread use in selecting aptamers for various applications, including bacterial detection [98].

The intercalated motif (I-motif) is a structure involving a hemiprotonated C•C^+^ base pair formed under slight acidic pH conditions (Figure 2C). Although several aptamers have C-rich sequences [67], only one aptamer has been shown to form an I-motif [99]. None have been developed in the context of bacterial detection. This may be explained by the lack of structural studies on aptamers or the extensive use of secondary structure prediction, which does not predict I-motif formation. Aptamers that form an I-motif for bacterial detection represent a potential area for future research. Prediction software has been overwhelmingly used to predict the secondary structures of aptamers. However, these predictions consider simple rules of base pairing and free energy and fail to predict other secondary structures, such as G-quadruplexes or I-motifs [100,101,102].

Different methods have been used to evaluate the K_d_, relying on either label-based or label-free techniques [103]. Label-based approaches, using fluorescent or radioactive labels, lower the detection limit in the experiments, resulting in the more accurate determination of the K_d_. Label-free methods, such as surface-based detection methods, are also often used. One of the most commonly used methods is surface plasmon resonance (SPR), an optical method used to detect molecular binding interactions between one mobile and one fixed molecule. SPR is used to evaluate different kinetic parameters, such as the association and dissociation rate constant and the dissociation/association constant [104].

These traditional methods have been adapted to evaluate the binding affinity of an aptamer when the target is known, such as proteins or toxins that can be isolated or produced in a purified form. However, in the cell-based SELEX method, the target(s) for the aptamer is unknown. This, in turn, complicates the measurement of the kinetic parameters because the number of targets and their concentrations are unknown. This uncertainty makes it challenging to measure the binding affinity accurately and necessitates complex protocols in terms of the detection sensitivity and apparatus, such as microfluidics. Several studies have estimated the K_d_ using fluorescence-activated cell sorting (FACS) and fluorescence spectrophotometry [55,105,106,107]. Both methods require fluorescently labeled aptamers and cannot be performed in a multiplexed format.

Comprehensive descriptions of aptamers, including details on their thermal stability, topology, and structure, are essential for their effective utilization in various applications and in ensuring their effectiveness in real-life scenarios. The stability of nucleic acid structures can be evaluated through thermal denaturation experiments using either UV-CD absorption techniques or fluorescence techniques such as FRET [108,109,110]. Isothermal titration calorimetry (ITC) is used to derive the thermodynamic parameters and stoichiometry of the aptamer/target interactions. Several factors can influence the melting temperature of an aptamer, affecting its stability, such as the base composition, length, structure, ionic strength, and pH [111,112,113,114,115]. Moreover, the potential conformational changes in aptamers upon binding with their targets are rarely explored and deserve further investigation [116]. Resolving an aptamer’s high-resolution topology could lead to a better understanding of its target specificity and pave the way for the further optimization of its stability and specificity.

Various computational tools have been developed to complement the experimental characterization of aptamers. They can be applied in the preliminary stage of selection to complement SELEX, by designing high-affinity aptamers for the target, and in the final stage to optimize the aptamer’s implementation. The main limitation of in silico approaches lies in the limited experimental data on the structures of aptamer/target complexes [117]. Prediction methods have been used to obtain successful topological indicators, but experimental data are required to gain insights into the structure adopted by an aptamer.

Different experimental methods can be used to gain insights into the folding of aptamers. Shape experiments, which chemically probe unpaired or paired base pairs, are used to determine the secondary structures of nucleic acids [118,119]. These experiments are convenient for long sequences. Spectroscopic methods such as UV, circular dichroism, or fluorescence can be used to obtain information on the folding topology of a nucleic acid [120]. For example, CD is widely used for G-quadruplex or I-motif structures to gain insights into the G-quadruplex topology [121]. These relatively easy methods can be used to examine the dependence of the secondary structure, such as the ion type and concentration, pH, and buffer composition. This is particularly true for I-motifs [122,123] and G-quadruplexes [124,125]. This information is crucial in establishing a protocol that ensures correct folding and the monitoring of their stability over time.

Three main experimental techniques have been used to resolve the structures of aptamers in free form or in complex with their targets: nuclear magnetic resonance (NMR), X-ray crystallography, and cryogenic electron microscopy (cryo-EM). All three methods can give rise to high-resolution structures (see [126] for a comprehensive analysis of aptamer structures). NMR uniquely investigates nucleic acids’ conformational states and folding through simple 1D experiments. It monitors structures such as I-motifs, G-quadruplexes, or hairpins, specifically through the imino-proton chemical shift [127]. These methods provide detailed information and are becoming crucial in understanding the aptamer-binding mode. One notable example is an RNA aptamer’s three-dimensional resolution, which was used to recognize the bacterial protein Hfq of *Bacillus subtilis* [128]. Recently, there has been growing interest in resolving these aptamers’ complex structures [126,129,130].

Aptamers’ chemical modification and optimization are being increasingly explored, serving as a crucial strategy to enhance their binding affinity, thermal stability, and protection against nucleases. These properties directly influence the overall suitability of aptamers for biosensing applications. One significant disadvantage of aptamers as oligonucleotides is their high sensitivity to nuclease degradation, which affects the detection in biological samples, especially in serum [131]. Temperature fluctuations can also impact aptamers’ binding capacity and susceptibility to enzymatic degradation by nucleases. To overcome these limitations, various chemical strategies have been devised to thermally stabilize, protect against degradation, and enhance the binding affinity of the active conformation of an aptamer.

Chemical modifications to the base, phosphate group, or sugar unit have been used to enhance aptamers’ suitability [132,133,134]. For instance, the incorporation of non-natural phosphorothioate into the aptamer’s backbone structure and the modification of the 5′-ends or sugar structures of strands were shown to slow down the hydrolysis of nucleases present in biological fluids while retaining their activity [135,136,137,138]. Another approach to avoiding the problems related to aptamer degradation is the incorporation of non-natural base pairs (such as 5-methyl-isocytidine) or the utilization of L-DNA to make strands highly resistant to nuclease attacks [139]. The majority of such chemical modifications aim to improve the stability of nucleic acid structures that are highly vulnerable to nuclease digestion, particularly in single-stranded segments and internal nicks.

Aptamers can acquire relative nuclease resistance when covalently linked to a protective group such as a protein. For instance, a DNA-type thrombin-binding aptamer was shown to resist a variety of serum nucleases when it was tightly bound to its target protein [140]. Moreover, different protective coatings of DNA strands increase their nuclease resistance. Lacroix et al. increased the stability of a DNA strand by coating it with human serum albumin and DNA dendrites [141]. In another study, an oligolysine–polyethylene glycol (PEG) coating was shown to protect DNA structures from low-salt denaturation and nuclease degradation [142]. The stability can also be improved via the ligation of strand termini or the introduction of terminal functional groups, such as hexanediol or hexaethylene glycol; through the crosslinking of nucleic strands via click chemistry; or through the formation of a thymidine dimer using ultraviolet irradiation [143,144]. These chemical modifications eliminate internal nicks once they are constructed. In a different approach, El-Khoury and Damha used chemical end-ligation to stabilize an intramolecular I-motif at both an acidic and neutral pH using deoxy-2-fluoroarabinocytidine [145]. This method allows for the use of I-motifs on a large scale for in vitro applications such as detection.

Finally, the effect of nucleases on the aptamer structure can also be minimized by applying conditions to denature nucleases or by using nuclease inhibitors. For instance, detection in serum can be performed after serum heat pretreatment at 75 °C for 10 min, thus rendering most nucleases inactive [146].

## 4. Aptasensors for Bacterial Detection

**Figure 3:**
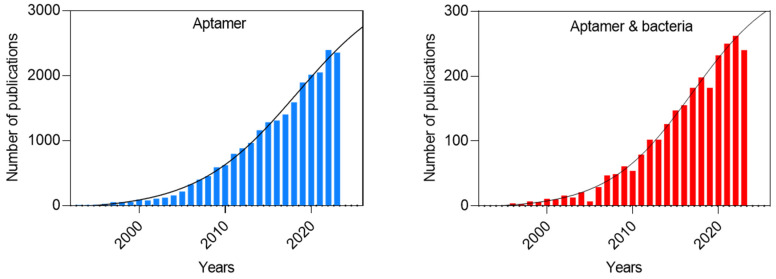
Aptamer development and their application in bacteriology in the literature in the period of 1990–2023 Values were obtained by searching “aptamer” and “aptamer & bacteria” keywords in Scopus. Trends obtained by fitting a tendency curve and projecting it for the next 4 years.

Given the increasing prevalence of foodborne and infectious diseases, many aptamers have been selected and optimized to target bacteria, as illustrated in Figure 3. Biosensors that integrate aptamers as recognition elements are usually called aptasensors. The detection potential of aptamers plays a pivotal role in these biosensors’ analytical performance. Fortunately, aptasensors can satisfy the REASSURED criteria (Affordable, Sensitive, Specific, User-Friendly, Rapid, Equipment-Free, Delivered, Real-Time, and Ease of Specimen Collection and Environmental Friendliness) established by the WHO for high-quality diagnostic devices that can be used across all levels of the healthcare system [147].

Aptamers possess a high binding affinity, comparable to that of monoclonal antibodies, and have several advantages over expensive antibodies when used in biosensors [148]. The primary advantages include their structural complexity and the possibility of fine-tuning through post-SELEX modifications, enabling the enhancement of their binding affinity and biochemical stability in complex matrices [149,150]. Moreover, in contrast to antibodies, aptamers provide ease of production due to their small sizes and have low immunogenicity [151,152,153]. Finally, these small nucleic acid molecules have lower synthesis costs compared to antibodies and can be easily customized to accommodate changes [154].

Aptasensors composed of aptamers that can be modified with an optical or redox reporter at one terminus and attached to the sensor via the other end transform the recognition of targets into a measurable signal that can be electrochemical, optical, or mass-sensitive. Moreover, some aptasensors combine different techniques; for example, a dual-mode sensing platform for the detection of *Clostridium perfringens* was obtained that combined electrochemical signals and fluorescence [155].

### 4.1. Electrochemical Aptasensors for Bacterial Detection

Electrochemical aptasensors have immobilized aptamers on the working electrode, allowing for the conversion of the recognition event into a measurable electrical signal. Since the first reported electrochemical aptasensor for thrombin detection in 2004, devices have been developed that couple the use of aptamers with different electrochemical methods, such as amperometry (where a measurable current intensity is generated), potentiometry (where a potential is measured while keeping the current constant), voltammetry (where a current change is measured under controlled potential variations), and impedance (where a relationship between the alternative current and the applied sinusoidal potential in the frequency domain is measured) [156,157]. Electrochemical aptasensors enable bacterial detection in very small volumes of several µL, with high sensitivity.

Usually, thiolated DNA aptamers are employed because they can be covalently self-assembled onto a gold electrode. To provide robust and reproducible results, thiolated aptamers should be reduced to break the disulfide bonds between the aptamer molecules immediately before immobilization on a clean gold surface [158]. Alternatively, the electrode surface can be modified using nanomaterials to increase the active electrode surface, which in turn enables one to enhance the signal [20]. Compared to other analytical methods, such as chromatography or mass spectroscopy, electrochemical methods do not require sophisticated equipment or complex sample preparation steps and are affordable. Finally, electrochemical biosensors can be easily fabricated “in-house” and miniaturized into a hand-held format. Table 1 summarizes the recently reported aptamer-based electrochemical biosensors for the detection of bacterial cells or their toxins.

Infectious bacterial agents differ in numerous aspects, including their virulence factors, contagion, and transmission. All these factors and molecules, playing a role in the bacterial lifecycle, can be targeted by aptamers to enable specific and efficient bacterial detection and identification using aptasensors. There are significant practical advantages to the development of aptasensors for the detection of whole bacterial cells. The diversity of biomarkers and their abundance on a cell surface provide the potential for higher sensitivity than when targeting a single protein expressed at a low level. In addition, the targeting of whole cells facilitates the overall detection procedure, as the sample preparation stage prior to analysis is highly simplified [159]. In most traditional and molecular methods for bacterial detection, sample preparation is the most expensive and the most time-consuming step. Finally, the selection of specific aptamers is also facilitated because there is no need for the lengthy process of purifying a specific target biomarker [160]. However, bacterial surfaces can vary in structure and composition depending on the environmental conditions. This bacterial adaptation to external conditions may modify their surface molecule compositions, which influences the reliability of diagnostic tests. This problem can be overcome by the utilization of a mixture of aptamers that target different surface epitopes of the target bacterium [161,162].

**Figure 4:**
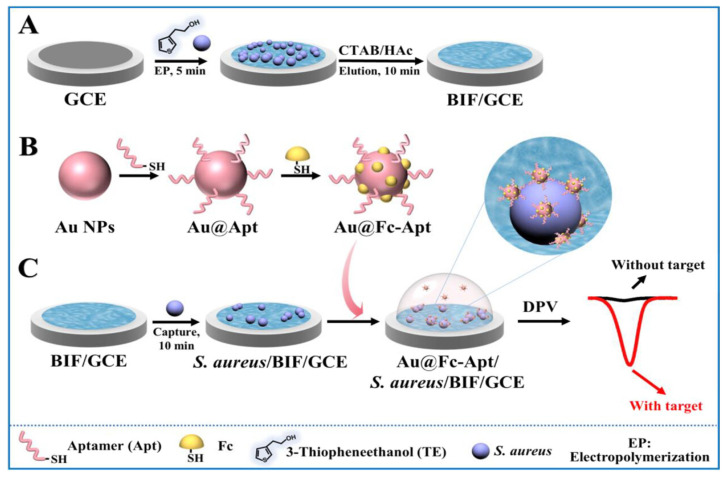
Schematic illustration presenting (**A**) preparation of the BIF-modified glassy carbon electrode, (**B**) preparation of aptamer carrying signal nanoprobe, and (**C**) dual BIF-aptamer based electrochemical sandwich detection of *S. aureus* cells. Reprinted with permission from [163]. Copyright (2023). American Chemical Society.

A recently developed bacterial electrochemical aptasensor was designed for the selective and ultrasensitive detection of *S. aureus* at the single-cell level using a sandwich assay [163]. The aptasensor relied on dual recognition through a bacteria-imprinted polymer film (BIF) and an aptamer (Figure 4). The BIF, which served as a capture probe, was fabricated on a glass carbon electrode, whereas the aptamer, which served as a detection probe, was functionalized with 6-ferrocenyl-hexanediol on gold nanoparticles (AuNPs). The aptamer was produced with a thiol on its 5′-end to allow its covalent binding to the AuNPs. After anchoring *S. aureus* on the BIF-modified electrode, the AuNP–aptamers were introduced to bind the captured bacteria, leading to an amplified current signal. The achieved ultrasensitive detection enabled the sensing of a single *S. aureus* cell in a buffer. The limit of detection (LoD) of the aptasensor was 10 CFU/mL when used in complex, lipid-rich media such as milk. This high sensitivity suggests the sensor’s applicability in food safety and prevention.

**Figure 5:**
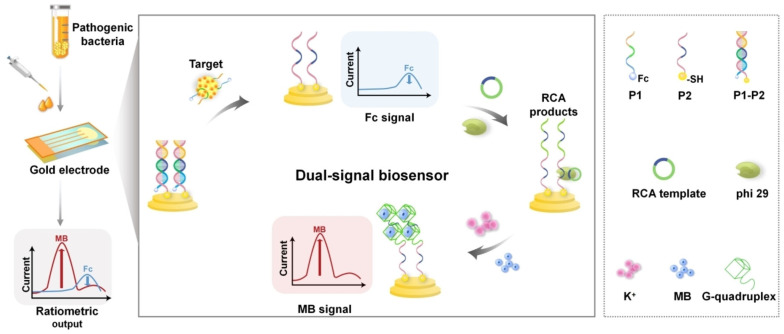
Schematic illustration presenting ratiometric dual-signal electrochemical biosensor for ultrasensitive detection of pathogenic bacteria based on aptamer recognition-induced rolling circle amplification (RCA)/G-quadruplex strategy. Adapted with permission from [164]. Copyright (2023) Wiley.

In another recent example, Ding et al. [164] developed a novel ratiometric dual-signal electrochemical aptasensor for pathogenic bacteria at the single-cell level. This sensor was based on the aptamer-recognition-induced rolling circle amplification/G-quadruplex strategy [165,166,167]. G-quadruplexes can interact with various signaling molecules, including the electrochemical signal redox indicator methylene blue (MB). In this study, signal amplification was achieved through the utilization of two probes—one being an aptamer modified with ferrocene for the target bacteria and the other being the primer sequence for rolling circle amplification (RCA), anchored to the gold electrode with a sulfhydryl probe (Figure 5). These two probes formed a complex that was disrupted by the presence of the target bacterium, *S. aureus*. Consequently, when contaminated samples were tested, only the RCA probe remained on the gold surface. To stimulate the RCA reaction, a C-base-rich circular template was introduced to the reaction mixture, generating a multitude of G-base-rich nucleic acid sequences that formed the RCA products into a G-quadruplex. Upon the addition of MB, the substantial cascade amplification of the electrochemical signals occurred due to the strong affinity of the G-quadruplex [168].

**Figure 6:**
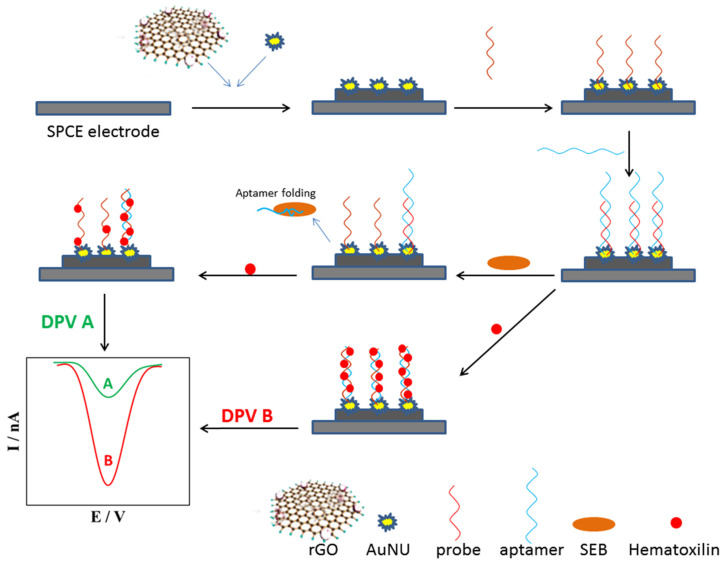
Schematic illustration of fabrication process of SEB aptasensor using reduced graphene oxide and gold nano-urchins. Adapted with permission from [169]. Copyright (2019) Elsevier.

An electrochemical aptasensor was also designed for the detection of staphylococcal enterotoxin B (SEB), a potent bacterial toxin responsible for severe food poisoning [169,170]. The sensor utilized a commercial carbon screen-printed electrode modified with reduced graphene oxide (rGO) and gold nano-urchins (AuNUs). The electrode was further functionalized with a single-stranded DNA probe, followed by the attachment of a specific aptamer. Upon encountering SEB molecules, the aptamer detached from the electrode surface. Following this, the electrochemical signal of the redox probe, hematoxylin, decreased significantly (Figure 6). The aptasensor’s analytical performance was validated with food samples, serving as simulated real samples. This tool surpassed a commercial ELISA kit in terms of SEB detection. Employing this method, the potential application of the aptasensor to food sample screening was demonstrated.

**Figure 7:**
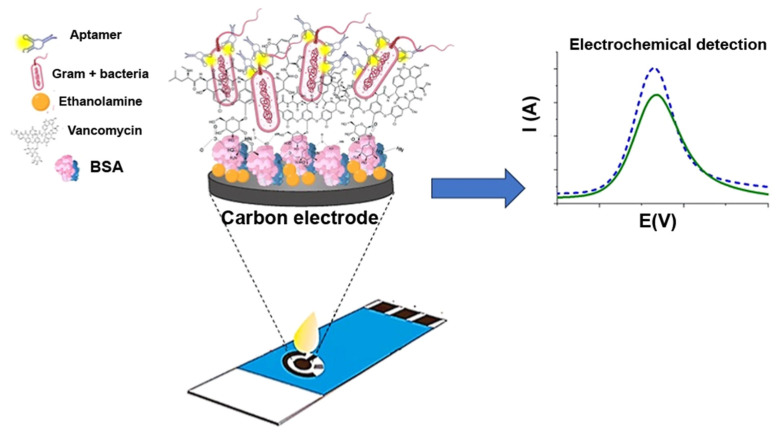
Schematic illustration of vancomycin/aptamer sandwich electrochemical detection of *S. aureus* and *B. cereus*. A screen-printed carbon electrode (SPCE) was modified by drop-casting with bovine serum albumin (BSA). Vancomycin was attached to the surface, and ethanolamine was used as a blocking agent. When bacterial suspension was added to the surface, only Gram-positive bacterial cells were attached. In the last step, the captured bacterial cells were identified by specific aptamers. Adapted with permission from [171]. Copyright (2024) American Chemical Society.

Aptamers can be combined with other sensing elements to improve the specificity and sensitivity of bacterial detection. For instance, the selective dual-aptamer–antibiotic-based sandwich detection of *S. aureus* and *B. cereus* was recently developed [171]. In the system, vancomycin, which binds to the cell walls of Gram-positive bacteria, was immobilized onto a carbon electrode. This electrode concentrated the bacterial cells in less than 10 min in a highly efficient manner, as shown using impedance measurements. The captured bacterial cells were then identified using a strain-specific aptamer and differential pulse voltammetry (Figure 7). When applied directly in milk or bovine serum, the aptasensor identified *S. aureus* and *B. cereus* with an LOD of 100 CFU/mL in less than 45 min.

**Figure 8:**
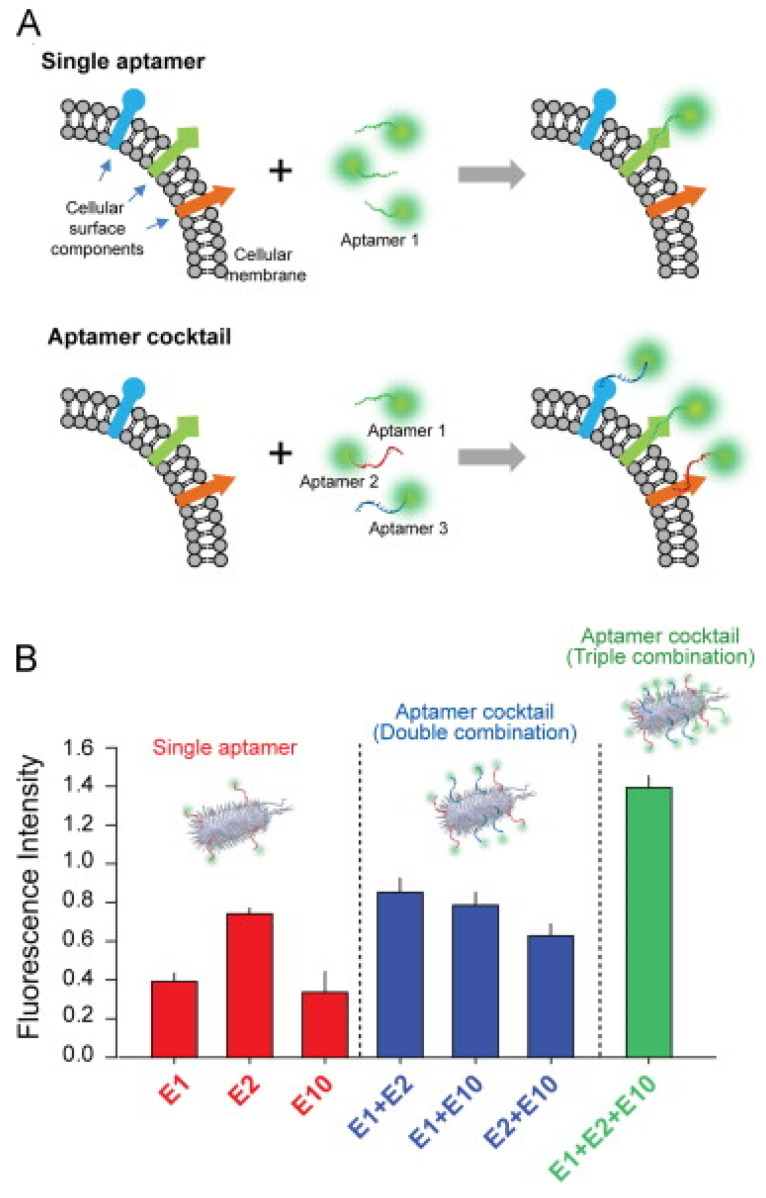
(**A**)Schematic illustration of detection signal enhancement by a mixture of three aptamers for *E. coli* detection. (**B**) Intensities of fluorescence obtained by staining bacterial cell with single, double, and triple aptamers. Adapted with permission from [162] Copyright (2014) Elsevier.

Alternatively, the robustness of bacterial detection can be improved by using a mixture of different aptamers targeting the same bacterium (Figure 8A). Kim et al. showed the signal enhancement and improvement in sensitivity obtained through the combinatorial use of aptamers immobilized on gold electrodes. The detection limit for *E. coli* achieved using the aptamers individually was approximately 18 times that obtained when the three aptamers were used in combination, as demonstrated using cyclic voltammetry [162]. This high efficiency in *E. coli* detection was confirmed by the fluorescent signal observed with the mixture of the three aptamers, which was higher than that obtained for any single aptamer (Figure 8B). This enhancement likely resulted from the presence of numerous binding epitopes on the surface of a single bacterial cell.

### 4.2. Optical Aptasensors for Bacterial Detection

Optical aptasensors are among the most studied devices due their relatively easy utilization and high sensitivity, robustness, reliability, and potential to be integrated on a single chip. Optical transducer technologies include plasmon surface plasmon resonance (SPR), optical fibers, localized SPR (LSPR), surface-enhanced Raman scattering (SERS), and fluorescence-based and colorimetric sensors. These optical techniques allow bacterial detection with non-labeled aptamers. Some representative, recently developed aptamer-based optical biosensors are shown in Table 2.

Plasmonic sensors, such as SPR, LSPR, and SERS, employ gold or silver metallic thin films and nanostructures due to their higher optical absorption bands in the visible–near-infrared range of the electromagnetic spectrum, called the plasmonic band. SPR couples the resonance of the evanescent field of transverse magnetic polarized light with an oscillating free electron wave bound at the metallic–dielectric interface. A conventional prism with a Kretschmann configuration can be used to achieve SPR-based biosensors for quantitative target detection. Although very sensitive, SPR sensors based on noble metal substrates suffer from low penetration depths (100–300 nm) into the dielectric medium above the surface, preventing the detection of bacterial cells of several µm. However, recently, portable SPR biosensors were developed for the monitoring of bacterial biofilms by coupling an insulator–metal–insulator structure with a higher penetration depth in the Kretschmann configuration [172].

When metallic nanostructures are used, the resonant field is associated with surface plasmons, which is referred to as LSPR. In these biosensors, the resonance wavelength depends on the shape and size of the plasmonic nanoparticles and the surrounding medium. Based on this, LSPR aptasensors have been realized through aptamers’ covalent or non-covalent immobilization on plasmonic nanoparticles. Kim et al. developed a label-free LSPR aptasensor for the detection of *Campylobacter* in contaminated chicken carcasses [173]. The specific aptamers were simply adsorbed onto gold nanoparticles (AuNPs) and then mixed with the water used to rinse chicken carcasses. In the absence of the pathogen, the AuNPs were stable and showed a plasmonic band at 520 nm. However, in the presence of *C. jejuni* or *C. coli* cells, the aptamers desorbed from the AuNPs to bind to the target, causing the ion-induced aggregation of the AuNPs. The aggregated AuNPs showed a plasmonic band at 630 nm. The aptasensor was able to detect *Campylobacter* in chicken carcasses after a two-day enrichment period, with an LOD of 7.2 × 10^5^ CFU/mL for *C. jejuni* and 5.6 × 10^5^ CFU/mL for *C. coli*. A similar approach was used to detect *S. aureus* cells in milk and infant formulas [174]. Due to the use of nanoparticles instead of conventional prisms, LSPR biosensors possess a high surface-to-volume ratio for the sensing of surface–bacterial interactions. This enables the use of miniaturized LSPR devices with applicability for in-field measurements. Moreover, when only qualitative bacterial detection is sufficient, LSPR aptasensors provide instrument-free result visualization. The main disadvantage of these biosensors is their low sensitivity. The sensitivity of SPR and LSPR sensors can be improved by coupling them with enzymatic reactions or by labeling the aptamers with a fluorophore.

In SERS devices, the Raman signal can be enhanced by placing a bacterial cell within the localized electric field of the plasmonic nanoparticles to increase the Raman scattering cross-section and hence the detection signal [175]. SERS has high selectivity and can provide a single bacterial cell’s detection due to the molecular fingerprint information. In addition, it is a fast and nondestructive method and can be used for in-field multiplex qualitative analysis. Bacterial cells are usually negatively charged and can be non-specifically immobilized on positively charged SERS substrates, as shown for *Salmonella typhimurium* [176]. However, aptamers can be used to specifically capture and immobilize a target bacterial cell to improve the specificity and selectivity of detection. For instance, an SERS-based aptasensor for the quantification of *Salmonella typhimurium* was designed by combining a Raman-active molecule, p-aminothiophenol (PATP), gold nanorods (GNRs), and cDNA, to which the aptamer could bind. In the absence of the target bacterium, the *S. typhimurium* aptamer was bound to the GNRs and specifically interacted with the cDNA, which prevented particle aggregation in a salt solution [177]. However, in the presence of both *S. typhimurium* and cDNA simultaneously, the aptamer no longer stabilized the GNRs in the salt solution, leading to particle aggregation, which gave rise to the SERS signal reflected by the attached component. Furthermore, the SERS intensity was linearly proportional to the *S. typhimurium* concentration, ranging from 56 to 56 × 10^7^ CFU/mL, with an LOD of 9 CFU/mL. Due to the aptamer’s specificity, the aptasensor showed high selectivity to other bacterial pathogens, and it was able to screen adulterated milk samples for the presence of *S. typhimurium*. Despite the many advantages of SERS aptasensors, they suffer from the requirement for sophisticated instrumentation to provide reliable detection.

**Figure 9:**
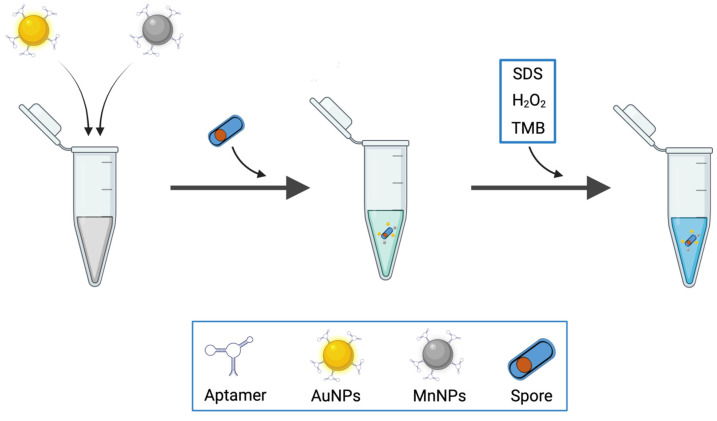
Schematic illustration presenting colorimetric biosensor for detection of *B. cytotoxicus* spores based on spore-enhanced peroxidase-like catalytic activity of AuNPs. Adapted with permission from [19].

Besides LSPR detection, colorimetric biosensors also provide the possibility for naked-eye bacterial detection. Bacterial spores remain a major concern in the food industry and hospitals as they are remarkably resistant to high temperatures, chemical agents, radiation, and harsh physical conditions. In their sporulated forms, bacteria exhibit resilience to unfavorable environmental conditions, making them the most resistant life forms known to date. Typically, they can withstand temperatures 40–45 °C higher than their corresponding vegetative cells. Additionally, spores can enter dormancy and survive in a wet state for thousands of years. Furthermore, spores are regarded as potential biological weapons. Indeed, *Bacillus anthracis* was initially used during the First World War. The dispersal of spores in the ambient air can result in the development of the respiratory form of anthrax. Due to these unique characteristics, in the context of biosecurity and food safety, researchers have developed aptasensors specifically designed to detect target bacteria in their sporulated forms [5,74,178]. Recently, Rizzotto et al. developed a colorimetric assay for the detection of *Bacillus cytotoxicus* spores in food [19]. *B. cytotoxicus* belongs to a large group of bacteria commonly named the *Bacillus cereus* group [6,162]. The group is composed of different species; some of them are human pathogens (such as *B. anthracis*, *B. cereus sensu stricto*, or *B. cytotoxicus*) but others are harmless (such as *B. thuringiensis*, *B. weihenstephanensis*, and *B. mycoides*). The detection test relied on the spore-enhanced peroxidase-like catalytic activity of gold nanoparticles (Figure 9). The test was performed in a simple microtube containing AuNPs and magnetic beads (MP), both conjugated with a specific aptamer, BAS6R, as a recognition element for *B. cytotoxicus* spores. The presence of spores was detected with the naked eye due to the change in the color of the solution (blue to dark blue) upon the oxidation of tetramethylbenzidine (TMB) with H_2_O_2_. This method has the advantage of the aptamer binding to an unknown epitope on the spore surface, eliminating the need to extract, purify, and amplify a specific biomarker. However, BAS6R was shown to bind to spores of different *B. cereus* species. It cannot thus distinguish between pathogenic and non-pathogenic strains of this group of bacteria.

In an another study, Zhou et al. developed a dual-aptamer microfluidic chip for the detection of both vegetative cells and spores of *B. cereus* [179]. To improve the selectivity and sensitivity of detection, aptamers selected through cell-SELEX were additionally, in a step-by-step manner, tailored and optimized using molecular docking. Based on this dumbbell aptamer design, the two best aptamers were obtained and integrated into a microfluidic chip biosensor. These two aptamers had repeated G-based structures, both with a strong affinity to the polar amino acids in the α-helix of the epiprotein of *B. cereus*. The biosensor was applied directly in food, such as milk and rice; provided a complete analysis within 1 h; and showed an LoD of only 9.27 CFU/mL.

As mentioned before, aptamers’ structures are susceptible to matrix interference, which diminishes their ability to specifically bind to target bacteria and affects the specificity and accuracy of aptasensors. To avoid a high rate of false negatives when *Salmonella* was detected in food samples, Zhao et al. studied the dual-recognition effect of an aptamer and concanavalin A [180]. Concanavalin A bound to the lectin in the Gram-negative bacterium. The method consisted of the separation of *S. typhimurium* via aptamer-functionalized magnetic beads, and the salt-induced aggregation of concanavalin A protected the gold nanoparticles (AuNPs). The developed visual method had improved specificity and sensitivity due to the targeting of different of sites at the bacterial envelope.

### 4.3. Other Aptasensors for Bacterial Detection

Detecting bacteria is not always sufficient. Many bacterial pathogens, such as *S. aureus*, *E. coli*, *C. botulinum*, *C. perfringens*, *C. difficile*, *B. cereus*, *C. jejuni*, *L. monocytogenes*, *Yersinia enterocolitica*, and *Salmonella* spp., produce toxins [181,182,183,184,185,186,187,188,189]. Ingested bacteria toxins have various effects, ranging from disrupting cell membranes and causing cell death to interfering with the host immune response. They play a crucial role in the pathogenicity of many bacterial species, contributing to the symptoms and severity of bacterial infections [190]. For instance, *C. botulinum* toxins can cause botulism, a severe illness characterized by muscle paralysis, general weakness, and potentially fatal respiratory failure. The presence of *S. aureus* enterotoxins in food can lead to symptoms such as nausea, vomiting, and severe diarrhea. As already mentioned, these toxins are thermostable and can rarely be eliminated by food treatment or preparation methods such as cooking, frying, or freezing, which can eliminate bacterial cells. Moreover, toxins can remain undetected by classical methods of pathogen detection. Usually, when strains are isolated, the presence of toxins is tested via the PCR amplification of the genes that encode them. This additional step prolongs the procedure and cannot be performed in the field. In addition, the presence of genes does not necessary lead to toxin production. Thus, there is a need to directly detect the presence of bacterial toxins in food and biological samples. Some representative, aptasensors are shown in Table 3

**Figure 10:**
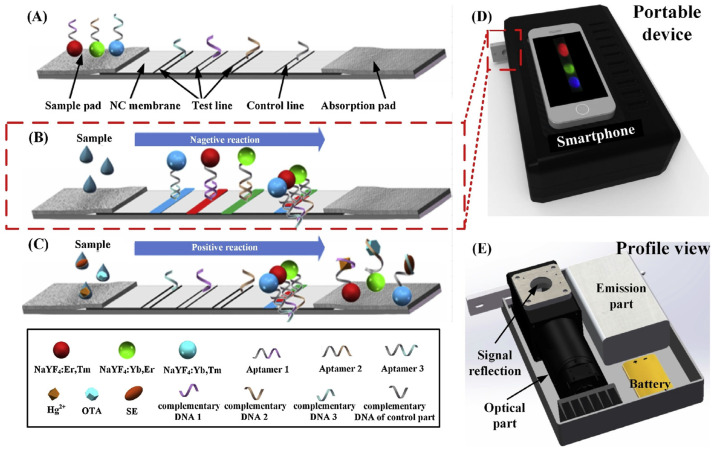
Schematic illustration of lateral flow assay based on three different aptamers for simultaneous multiple targets detection. (**A**) sample pad design, sample pad in (**B**) negative and (**C**) positive reaction, (**D**) front and (**E**) profile view of the device. Adapted with permission from [191]. Copyright (2018) Elsevier.

To assess food safety, multiplex aptasensors have been developed for the detection of different targets in food samples. Jin et al. proposed a lateral flow strip (LFT) aptasensor for *Salmonella*, Hg^2+^ ions, and ochratoxin A (OTA) using upconversion nanoparticles (UCNP) as a signal source and a smartphone equipped with a CCD camera for detection [191]. This approach offers a fast and portable detection process. As illustrated in Figure 8, three different aptamers were used in the sensor, each grafted on the surfaces of differently colored UCNPs and hybridized with their complementary DNA (cDNA) fixed on the nitrocellulose membrane. In the presence of bacteria, ions, and small molecules, the three aptamers preferentially bound to their respective targets. Consequently, the liberated cDNA decreased the fluorescence from the UCNP. It was demonstrated that this device had no non-specific or cross-reactivity due to the separate color channels providing the detection signals. This feature was crucial to enable multiplexing. This method of detection holds great potential for the simultaneous detection of multiple targets in food samples to achieve enhanced safety (Figure 10).

A bacterial biological marker or bacterial biomarker can be defined as a specific characteristic that is measured as an indicator of a bacterium’s presence, its biological processes, or its responses to external exposure or intervention, including therapeutic interventions. The detection of biomarkers enables various applications, such as the monitoring of infection and therapy efficiency, risk estimation, diagnosis, or the estimation of bacterial adaptation to environmental signals. Numerous aptamers have been developed to target a wide array of bacterial biomarkers, although targeting the entire cell is more common. Some aptamers have been developed for purposes other than detection, such as therapeutic or basic research applications. This technology has paved the way for new applications that are not only cost-effective but also capable of overcoming the limitations posed by conventional antibodies or various probes and drugs.

The methyl erythritol phosphate (MEP) metabolic pathway of isoprenoid biosynthesis is essential for many pathogenic bacteria, both Gram-positive and Gram-negative, such as *B. anthracis*, *Clostridium* spp., *L. monocytogenes*, *S. enterica*, *Vibrio cholerae*, *Shigella* spp., *E. coli*, and *Pseudomonas aeruginosa*. Recently, a DNA aptamer, D10, was developed to target 1-deoxy-D-xylulose-5-phosphate reductoisomerase (DXR), the second enzyme of the MEP pathway [192]. This aptamer was modified with a fluorescent label and allowed the strong, specific staining of MEP+ bacterial cells (*E. coli* and *P. aeruginosa*), while no staining was observed with bacteria lacking the DXR enzyme (e.g., *E. faecalis*). It thus presents the potential to replace the specific antibody for future therapeutic and diagnostic applications.

More recently, an antagonist aptamer for SEB neutralization was selected through SELEX [193]. SEB is a critical virulence factor in staphylococcal toxic shock syndrome (TSS), triggering the robust release of proinflammatory cytokines by activating T lymphocytes. The aptamer demonstrated effectiveness in inhibiting SEB-induced proliferation and cytokine secretion in human peripheral blood mononuclear cells. These results highlight the potential therapeutic value of the novel aptamer antagonist in mitigating SEB-mediated TSS, and it represents a promising avenue for future therapeutic strategies.

Another expanding aptamer application was described by Stoltenburg and his co-workers [98], who explored an alternative approach to develop aptamers that bind a bacterial surface protein. In contrast to cell-SELEX, which selects specific aptamers that bind unknown targets at the bacterial cell surface, their procedure was based on selecting aptamers that recognize a purified surface protein of *S. aureus*, called protein A. This protein is a major component of the cell wall of *S. aureus*. The obtained protein-A-binding G-quadruplex aptamer was integrated into an enzyme-linked oligonucleotide assay (ELONA). They demonstrated that the aptamer and its truncated versions bound to protein A expressed by the intact cells of *S. aureus*. Some structural information about the aptamer used was also gathered because the aptamer’s functionality was shown to be tightly linked to its structure. Such insights into the structural basis of aptamers’ functionalities enhance our understanding of the molecular bases of their interactions and pave the way for the development of novel, more robust aptamer-based diagnostic tools.

Many bacterial virulence factors are exported by external vesicles. All Gram-negative bacteria secrete outer membrane vesicles (OMVs), proteoliposomes derived from their outer membranes, with sizes between 20 and 250 nm. OMVs play crucial roles during host–microbe interactions because they transport a broad range of molecularly diverse cargo, including adhesins, toxins, membrane-embedded and -associated proteins, small molecules, lipids, peptidoglycans, and nucleic acids. Shin et al. selected aptamers that bound the OMVs of multiple Gram-negative bacteria [194]. The aptamers, selected through cell-SELEX, showing the strongest affinity to bind Gram-negative bacterial cells were conjugated with horseradish peroxidase (HRP) and used in an enzyme-linked aptamer essay (ELAA) to detect the OMVs. An LOD of 25 ng/mL was obtained. The recognition of the OMVs was expected because secreted vesicles contain similar proteolipids to their cells of origin. The authors suggested that the detection of bacterial OMVs was more effective than the detection of bacterial cells in clinical samples.

## 5. Conclusions

In the realm of pathogenic bacteria, biosensors developed for their rapid and accurate identification and monitoring offer important added value in preventing their spread, reducing treatment expenses, and ensuring food safety. Biosensors offer numerous advantages compared to traditional microbiological methods due to their enhanced sensitivity and rapidity. Currently, the identification of a microorganism typically takes between one and several days when plating or PCR-based protocols are employed. In contrast, biosensors may provide a complete microbiological analysis within several hours or even minutes [195,196]. Biosensors also hold the potential to be portable, multiplex, and accessible to a wide range of users [197,198].

Aptamers have been used extensively as an alternative to conventional antibodies in various biosensing strategies, including electrochemical, optical, and microfluidic chip-based platforms. Compared to antibodies, aptamers have a shorter production time (a few hours compared to a few days or even months), a lower cost (10- to 100-fold less expensive), higher stability in complex matrices, and the ability to bind a variety of specific targets, which enables their tailored application. However, the impact of replacing antibodies with aptamers in the lifecycle of biosensors remains to be examined. Regarding the latest research, trends in the use of aptasensors for bacterial detection include the development of portable biosensors that combine easy sample preparation with highly cost-effective biosensing strategies. Nonetheless, antibodies, which have been used for decades, are still far superior in terms of commercially available immunosensors. With this review, we aimed to demonstrate the potential of aptasensing in allowing the direct quantification of bacterial cells or toxins.

The specificity of detection and the accuracy of the overall analysis may be improved by the use of dual-aptamer probes, where the first aptamer acts as a capturing probe and the second is a detection probe. Moreover, a mixture of various aptamers, each targeting different epitopes of the same bacterium, can be employed to achieve higher accuracy in detection. To address the specificity limitations in aptasensors, the current research also considers the combination of aptamers and other sensing elements, such as vancomycin or concanavalin A, that also specifically bind bacterial cells.

Signal amplification strategies in aptasensors can be achieved by using different nanomaterials, catalysis amplification, hybridization chain reactions, and rolling circle amplification [20]. The extension and application of these strategies should considered in further surface chemistry investigations related to improvements in the aptamer’s immobilization onto the nanomaterial-modified sensor surface. The efficiency of nanostructured sensor surfaces to act as a filtering porous layer helps to eliminate the possible interference of the biological matrix and provides highly selective and sensitive detection. Moreover, aptamers adopting specific structural 3D conformations, such as G-quadruplexes, have been shown to provide high signal intensities (REF). The utilization of aptamers of specific 3D structures as recognition elements appears to be a smart and cost-effective approach to the development of sensitive biosensors [199]. At the same time, aptamers’ structural changes upon target binding may induce signal transduction and/or result in target-triggered amplification, which highly simplifies device development [200]. Considering that the primary sequences of many selected aptamers suggest that they adopt a specific 3D structure, it will be helpful to determine their secondary and tertiary structures for biosensor development. Indeed, a precise understanding of how aptamers interact with their targets will facilitate the design of more efficient and selective biosensors. This is especially relevant for aptamers adopting I-motifs (C-rich sequences) [67], which have still not been explored for bacterial detection.

## 6. Future Directions

Aptasensors require significant improvement to become a standard technique for microbiological applications. Indeed, there is a significant number of papers presenting proofs of concept, but there are only a few commercially available aptasensors that can be utilized at the level of the food industry or in hospitals. First, most SELEX procedures are performed using the same oligonucleotide pool, and not all selected aptamers are highly specific. Second, the selection and structural characterization of aptamers are usually performed under defined conditions, but their configuration and binding properties can be modified upon temperature, ionic strength, or pH variations, resulting in a significant reduction in their binding affinities. Consequently, although some aptasensors provide efficient bacterial detection in the buffer, they are inefficient when applied in biological or food samples. Therefore, it is highly important to characterize the individual aptamer sequences within environments that mimic real matrices. These studies should be completed with chemical modifications of the aptamers to stabilize the active conformation to ensure robust detection. Third, commercialization requires standards and standardized aptasensor specifications. Moreover, employing low-cost biodegradable materials or developing reusable aptasensors could significantly reduce the costs of bacterial detection. The future research directions should include fully automated aptasensors, integrating sample preparation, reaction chips, and signal generation, to decrease the assay time and improve the storage stability. Another important barrier in the application of aptasensors is aptamer degradation in biological matrices containing DNAse or RNAse enzymes. Nevertheless, the chemical modification of aptamer molecules can be performed to prevent such degradation, although this is rarely performed. Alternatively, to develop a highly stable aptasensor, a pragmatic approach could be to use a real matrix and/or a biosensor surface starting from the initial stage, i.e., the SELEX process.

We anticipate that aptamers, which can be both diagnostic and therapeutic elements, could facilitate the prevention of bacterial infections. In this double configuration, the detection of specific bacterial pathogens could trigger targeted antimicrobial drug release. The design of such systems for food safety or healthcare may be cyclical but requires a fundamental understanding of the aptamer/bacteria interactions. It is obvious that multidisciplinary approaches at the intersection of molecular biophysics, microbiology, and engineering will play pivotal roles in transitioning aptasensors from the lab to immediate, in-field detection.

**Table 1:** Aptamer-based optical biosensors for bacterial detection. Aptamer sequences are provided in Supplementary Data.

Bacterium	Aptamer	DNA or RNA	Target	K_d_ (nM)	LOD	Linear Range (CFU/mL)	Ref.
*S. aureus*	T1 T2 T3 A14	RNA & DNA	IsdA protein	2.2 ± 0.5 1.0 ± 0.3 0.7 ± 0.4 4 ± 2	113 pM 17 pM 11 pM 485 pM	/	[56]
*S. aureus*	SH-Apt_2_	DNA	Whole cell	210.7	/	/	[61,201]
*S. aureus*	SA20 SA23 SA34 SA31 SA43	DNA	Whole cell	70.86 ± 39.22 61.50 ± 22.43 72.42 ± 35.23 82.86 ± 33.20 210.7 ± 135.9	/	/	[65]
*S. aureus*	Apt1 Apt2	DNA	Whole cell	35 129	7.5–8.4 × 10^4^ CFU/mL	10^4^–10^8^	[174,202]
*S. aureus*	H1 H2 cApt	DNA	/	/	10^1^ CFU/mL	10^2^–10^6^	[203]
*S. aureus*	A15	DNA	Enterotoxin A protein	48.57	8.7 × 10^−3^ µg/mL	0.01–10 µg/mL	[204]
*S. aureus*	H1 H2	DNA	Whole cell	/	4–8 CFU/mL	45–4.5 × 10^6^	[205]
*E. coli*	SH-Apt_1_	DNA	Whole cell	25.2	/	/	[55,123]
*E.coli*	E1 E2 E3	DNA	Whole cell	/	3.7 × 10^2^ CFU/mL	/	[162]
*E.coli*	/	DNA	Whole cell	/	45 CFU/mL	10^2^–10^8^	[206]
*E. coli*	/	DNA	Whole cell	/	0.05 CFU/mL	0.1–10^4^	[207]
*P. aeroginosa*	F23	DNA	Whole cell	17.27 ± 5.00	10^4^ CFU/mL	/	[208]
*B. cereus*	/	DNA	Whole cell	/	22 CFU/mL	49–49 × 10^6^	[209]
*B. cereus*	/	DNA	Whole Cell	/	4 CFU/mL	20–2 × 10^8^	[210]
*Acinetobacter baumannii*	AB K2	DNA	Whole cell	5.377 6.8	10 CFU/mL	10–10^5^	[211]
*Klebsiella pneumoniae*	K2	DNA	Whole cell	/	10 CFU/mL	10–10^5^	[211]
*Leptospira interrogans*	LAP3	DNA	Outer, embrane protein	133.13	57 CFU/mL	60–6 × 10^5^	[212]
*Listeria monocytogenes*	A8	DNA	Internalin A	/	10^3^ CFU/mL	10^3^–10^5^	[213]
*Salmonella*	Multi-apt	DNA	Multi	11.72	7 CFU/mL	10–10^7^	[214]
*Yersinia enterocolitica*	/	DNA	Whole cell	/	3 CFU/mL	10–10^9^	[215]
*Bacillus cytotoxicus*	BAS6R	DNA	Spore	/	10^2^–10^4^ CFU/mL	10^3^–10^4^	[19]
*Clostridium difficile*	No name	DNA G-quadruplex	Toxin A protein (TOA)	/	1 nM	0–200 ng/mL	[216]
*S. typhimurium*	STA	DNA	Whole cell	/	9 CFU/mL	56–56 × 10^7^	[177]
*S. typhimurium*	H2	DNA	Whole cell	/	4–8 CFU/mL	36–3.6 × 10^6^	[205]
*S. Typhimurium*	Apt ST	DNA	Whole cell	10	30 CFU/mL	10^2^–10^6^	[217]
*Vibrio parahaemolyticus*	Apt VP	DNA	Whole cell	16.88	10 CFU/mL	10^2^–10^6^	[217]
*Campylobacter jejuni*	ONS13 ONS-23TA	DNA	Whole cell	292.8 ± 53.1	7.2 × 10^5^ CFU/mL	/	[62,173]

**Table 2:** Aptamer-based electrochemical biosensors for bacterial detection. Aptamer sequences are provided in Supplementary Data.

Bacterium	Aptamer	DNA or RNA	Target	K_d_ (nM)	LOD	Linear Range (CFU/mL)	Ref.
*S. aureus*	Antibac1&2	DNA	Peptidoglycan	415 + 0.047 1261 + 0.280	82 pg/mL	/	[54,218]
*S. aureus*	Apt1	DNA	Whole cell	35	10–100 CFU/mL	10–10^5^	[163,171]
*S. aureus*	P1	DNA	Whole cell	/	10	10–10^6^	[164]
*S. aureus*	A-SEB	DNA	SEB protein	0.02	0.21 fM	5.0–500 fM	[169]
*E. coli*	ECA I ECA II	DNA	Outer membrane proteins (OMPs)	/	/	1 × 10^−7^–2 × 10^−6^ M	[219,220]
*B. cereus*	B15 B16	DNA	Whole cell	16.13 20.67	10 CFU/mL	/	[106]
*B. cereus*	13–18 13–24	DNA	Whole cell	22.75 36.72	9.27 CFU/mL	/	[179]
*Acinetobacterer baumanni*	/	DNA	Whole cell	/	150 CFU/mL	1 × 10^3^–1.0 × 10^8^	[221]
*Clostridium difficile*	/	DNA G-quadruplex	Toxin A protein (TOA)		1 nM	0–200 ng/mL	[216]
*Mycobacterium tuberculosis*	/	DNA	MPT64 protein	8.92	4.1 fMl	/	[222]

**Table 3:** Other aptamer-based biosensors for bacterial detection. Aptamer sequences are provided in Supplementary Data.

Bacterium	Aptamer	DNA or RNA	Target	K_d_ (nM)	LOD	Linear Range (CFU/mL)	Ref.
*S. aureus*	APT^seb1^	DNA	Staphylococcal enterotoxin B (SEB)	/	/	/	[51]
*S. aureus*	G1 #2 #18	RNA	Teichoic acid	/	/	/	[53]
*S. aureus*	H1	DNA	/	/	41 CFU/mL	4.1 × 10^1^ to 4.1 × 10^5^	[223]
*S. aureus*	AT-27 AT-33 AT-36 AT-49	DNA	α-toxin protein	/	/	/	[224]
*E. coli*	GN6 GN12	DNA	Outer membrane vesicles (OMV)	29.94 20.36	/	/	[194]
*E. coli*	6-3 8-1 8-7 8-8 8-12 8-13 8-19 8-35	RNA	Heme	188 309 256 371 445 425	/	/	[225]
*E. coli*	Stx1 stx2	DNA	Shiga toxin Viz, stx1, and stx2	47 pM 29 pM	44.5 pg/mL 41.3 pg/mg	50 pg/mL 100 ng/mg	[226]
*P. aeroginosa*	F23	DNA	Whole cell	17.27 ± 5.00	10^4^ CFU/mL	/	[208]
*Vibrio cholerae*	CT916		Cholera toxin (CT)	48.5	2.1–2.4 ng/ml	0–10 ng/mL	[45]
*Clostridium perfringens*	/	DNA	Whole cell	/	1 CFU/mL	1–10^8^	[155]

## Supplementary Materials

The following supporting information can be downloaded at: https://www.mdpi.com/article/10.3390/bios14050210/s1, Table S1: Sequences of aptamer used in biosensors for bacterial detection.
