# Supplementary material for: Recent Advances in Aptamer-Based Biosensors for Bacterial Detection
Source: Biosensors (Basel). 2024 Apr 23;14(5):210. doi: 10.3390/bios14050210 (PMC11117931; doi:10.3390/bios14050210)
Supplement: Supplementary file 1 [file biosensors-14-00210-s001.zip › biosensors-2929208-supplementary.pdf]

Supplementary Data

Recent Advances in Aptamer-Based Biosensors for Bacterial Detection

Vincent Léguillier [1,2], Brahim Heddi [2,*] and Jasmina Vidic [1,*]

[1] INRAE, AgroParisTech, Micalis Institut, Université Paris-Saclay, UMR 1319, 78350 Jouy-en-Josas, France
[2] ENS Paris-Saclay, Laboratoire de Biologie et Pharmacologie Appliquée (LBPA), UMR8113 CNRS, 91190 Gif-sur-Yvette, France
* Correspondence: brahim.heddi@ens-cachan.fr (B.H.); jasmina.vidic@inrae.fr (J.V.)

Summary

- **Table S1.** Sequence of aptamers used for bacterial detection
- **References**

Table S1. Sequences of aptamer used in biosensors for bacterial detection.

Bacteria	Aptamer	Sequence	Ref
S. aureus	SA20	GCGCCCTCTCACGTGGCATCAGAGTGCCGGAAGTTCTGCGTTAT	[1]
	SA23	GGGCTGGCCAGATCAGACCCCGGATGATCATCCTTGTGAGAACCA	
	SA34	CACAGTCACTCAGACGGCCGCTATTGTTGCCAGATTGCCTTTGGC	
	SA31	TCCCACGATCTCATTAGTCTGTGGATAAGCGTGGGACGTCTATGA	
	SA43	TCGGCACGTTCTCAGTAGCGCTCGCTGGTCATCCCACAGCTACGTC	
S. aureus	T1	ACTGTCrGrCrGrCrArCrGrCrGrUrGrUrGrUrArGrUrArCrArCrArCrGrArUrCrGrCrGrCrGrCrArCrArArUrArU	[2]
	T2	ACTGTCrArArUrUrUrGrArArUrArUrArUrUrArGrUrGrCrGrCrGrCrCrGrUrArGrUrGrUrGrUrArArArArArUrU	
	T3	ACTGTCrArArUrUrUrGrArGrUrGrUrGrUrGrArUrCrArUrArUrArUrCrGrUrArGrCrGrCrGrCrUrArCrArArCrC	
	A14	ACTGTCCACACCGCAGCAGTGGGAACGTTTCAGCCATGCAAGCATCACGCCCGT	
S. aureus	H1	GCAATGGTACGGTACTTCCTCGGCACGTTCTCAGTAGCGCTCGCTGGTCATCCCACAGCTACGTCAAAAGTGCACGCTACT<br>TTGCTAA	[3]
S. aureus	H1	GCAATGGTACGGTACCCCTATGCGCATGTACCATTGCAGTTGTCAGAGAGCGA	[4]
	H2	GGTACA/Dabcyl/TGCGCATAGGGGTACCGTACCATACCCCTATGCGCA/FAM/	
	cApt	GTACCGTACCATTGCTAGCGTCTTCCCGTCCTT	
S. aureus	Apt1	TCCCTACGGCGCTAACCCCCCCAGTCCGTCCTCCCAGCCTCACACCGCCACCGTGCTACAAC	[5,6]
	Apt2	TCCCTACGGCGCTAACCTCCCAACCGCTCCACCCTGCCTCCGCCTCGCCACCGTGCTACAAC	
S. aureus	SH-Apt_2	GCAATGGTACGGTACTTCCTCGGCACGTTCTCAGTAGCGCTCGCTGGTCATCCCACAGCTACGTCAAAAGTGCACGCTACT<br>TTGCTAA	[7,8]
S. aureus	A15	AGCAG CACAGAGGTCAGATG(N40)CCTATGCGTGCTACCGTGAA-	[9]
S. aureus	APT^{seb1}	GGTATTGAGGGTCGCATCCACTGGTCGTTGTTGTCTGTTGTCTGTTATGTTGTTTCGTGATGGCTCTAACTCTCCTCT	[10]
S. aureus	A-SEB	TGCAGGATCCGGTATCCGTGCACACACACCCAACAACCAGCTGCCGCACCGGAGGAATTCTCGT	[11]
S. aureus	G1	UCCGAACAGCGGAAGGUGGUUCGAAGUUGGGGCUUUGGA	[12]
	#2	GGGAGUUUUGAUACGGCUUCAUGCAGUAAUGUUUUUAU	
	#18	UCCGAACAGCGGAAGGUGGUUCGAAGUUGGGGCUUUGGA	

S. aureus	AT-27<br>AT-33<br>AT-36<br>AT-49	ACCCCTGCAGGATCCTTTGCTGGTACC-(N42)-AGTATCGCTAATCAGTCTAGAGGGCCCCAGAAT	[13]
S. aureus	H1, P1	GCAATGGTACGGTACTTCCTCGGCACGTTCTCAGTAGCGCTCGCTGGTCATCCCACAGCTACGTCAAAAGTGCACGCTACT TTGCTAA	[14]
S. aureus	Antibac1<br>Antibac2	GGGACAGGGAGTGCGCTGCTCCCC<br>GGGGACTAGAGGACTTGTGCGGCC	[15,16]
E. coli	SH-Apt$_1$	GCAATGGTACGGTACTTCCCCATGAGTGTTGTGAAATGTTGGGACACTAGGTGGCATAGAGCCGCAAAAGTGCAGCTACT TTGCTAA	[17,18]
E. coli	/	ATCCGTCACACCTGCTCTATCAAATGTGCAGATATCAAGACGATTTGTACAAGATGGTGTTGGCTCCCGTAT	[19]
E. coli	GN6<br>GN12	ATACCAGCTTATTCAATTGGGTGAGGGGGGGTTCACAACGTTAAAGATAGACGGGGGAAGATAGTAAGTGCAATCT	[20]
E. coli	6-3<br>8-1<br>8-7<br>8-8<br>8-12<br>8-13<br>8-19<br>8-35	UGGUUUCAGCGACAGGAGGGGUGUAGGUGGAUUGCUGUCCUUUGCGUGU<br>UGCUAGUGUUGUAUGCACGUGGAGGAGGAGGCGUACACUUGCUUUGUGGU<br>GAUUGACCGUAUGGAGGAUGCAAAGGGAGGGAGGUCACUUGAGUUAGUUA<br>GCAGGAUGUGGAGGAGGCAUCUGCUGCAAUCGGGACUUGUGUCGAGUAUC<br>GCAUUGUCUGCGUGUGGAGGCAGGAGGCAAGAUAAGAGGUGAUGCGGUUG<br>CAUGUUGGCGAUACGUCUAAACGGUGGGUUGUGGAGGAUUGAUUUAUACG<br>AGUAGUGUCAGCGUGUGGUGGAGGUUGGCGACAUAUGUAGGGUGCGAUUG<br>UGCGCAAUACACGGUGAGGAGGUGGAGAGAUGUAGGUGCUUAGCAGUUGA	[21]
E. coli	ECA I<br>ECA II	GTCTGCGAGCGGGGCGCGGGCCCGGCGGGGGATGCGC<br>ACGGCGCTCCCAACAGGCCTCTCCTTACGGCATATTA	[22,23]
E. coli	/	GCAATGGTACGGTACTTCCCCATGAGTGTTGTGAAATGTTGGGACACTAGGTGGCATAGAGCCGCAAAAGTGCACGCTAC TTTGCTAA	[24]
E. coli	Stx1<br>stx2	ATCCAGAGTGACGCAGCAGTAGTTTGTTGGTTATTACGGCGGGTTGCGATGGGTGCGAATCGGTGGACACGGTGGCTTAG T<br>ATCCAGAGTGACGCAGCAGGAAAGGACGTCAAATTAGGGGCCGGGACAACGAAAGCCCACAACTGGACGGTGGCTTAG T	[25]

E.coli	E1<br>E2<br>E3	GCAATGGTACGGTACTTCCACTTAGGTCGAGGTTAGTTTGTCTTGCTGGCGCATCCACTGAGCGCAAAAGTGCACGCTACTTTGCTAA<br>GCAATGGTACGGTACTTCCCCATGAGTGTTGTGAAATGTTGGGACACTAGGTGGCATAGAGCCGCAAAAGTGCACGCTACTTTGCTA<br>GCAATGGTACGGTACTTCCGTTGCACTGTGCGGCCGAGCTGCCCCCTGGTTTGTGAATACCCTGGGCAAAAGTGCACGCTACTTTGCTAA	[26]
P. aeroginosa	F23	CCCCCGTTGCTTTCGCTTTTCCTTTCGCTTTTGTTCGTTTCGTCCCTGCTTCCTTTCTTG	[27]
B. cereus	/	AGCAGCACAGAGGTCAGATGCCCCCCTTTTATCCGTCGGCATGATGTCTCCCGATCCGGTCCTATGCGTGCTA	[28]
B. cereus	B15<br>B16	AGCAGCACAGAGGTCAGATGGGCGGGTTTGGATCTTTGGTTGGCGCCTGTTTCTTTATGACCTATGCGTGCTACCGTGAA<br>AGCAGCACAGAGGTCAGATGATATGTTTACGCCAGTGGTATTATTGGGGTTGATATGTCACCTATGCGTGCTACCGTGAA	[29]
B. cereus	13-18<br>13-24	AGCACAGAGGTCAGATGGGCTACTGGAGCATCTGGTAACGAAGTACCCTCGGGGCGG<br>AGCACAGAGGTCAGATGATCGAGGGCGCAGACCGAACCCGCGTGCGCAGTACAAGGGC	[30]
Acinetobacter baumannii	AB	AATCAGGCTCAGCATGGAGTTGCGAGGCCAATATCCGGTTAAGCG	[31,32]
Acinetobacterer baumanni	K2	ACAGCACCACAGACCACATATCACATGCTGTCGCCTTGCGATATCAATTCCAGTGATGTTTGTCTTCCTGCC	[31]
Leptospira interrogans	LAP3	TGGCGTTAGAGATACCGGAACCGGTGTCGGGCGTCTGAAGAATCC	[33]
Bacillus cytotoxicus	BAS6R	ATCCGTCACACCTGCTCTGCACGGGCTCAGTTTGGCTTTGTATCCTAAGAGGATGGTGTTGGCTCCCGTAT	[34]
Vibrio cholerae	CT916	GGCAAAAAGGATTGCCCAGGTCTGCTGTCTAGCCGGATTC	[35]
Clostridium difficile	/	TAGTGATGCCTTTGTTGAGA-[N40]-TCTTCATCGTCCACTAAATT	[36]
S. typhimurium	H2	TATGGCGGCGTCACCCGACGGGGACTTGACATTATGACAG	[14]
S. Typhimurium	Apt S.T	TGGCTAGCTCAGTCATATGGCGGCGTCACCCGACGGGGACTTGACATTATGACAGCCGCG	[37]

Vibrio parahaemolyticus	Apt VP	TGGCTAGCTCAGTCATCTAAAAATGGGCAAAGAAACAGTGACTCGTTGAGATACTCCGCG	[37]
Clostridium perfringens	/	TCAACGGCAGTAACATTAGC	[38]

1. Cao X, Li S, Chen L, Ding H, Xu H, Huang Y, et al. Combining use of a panel of ssDNA aptamers in the detection of Staphylococcus aureus. Nucleic Acids Research. 2009 Aug;37(14):4621–8.

2. Wijesinghe KM, Sabbih G, Algama CH, Syed R, Danquah MK, Dhakal S. FRET-Based Single-Molecule Detection of Pathogen Protein IsdA Using Computationally Selected Aptamers. Anal Chem. 2023 Jul 4;95(26):9839–46.

3. Lian Y, He F, Wang H, Tong F. A new aptamer/graphene interdigitated gold electrode piezoelectric sensor for rapid and specific detection of Staphylococcus aureus. Biosensors and Bioelectronics. 2015 Mar;65:314–9.

4. Kang Q, Xing X yan, Zhang S qi, He L, Li J zhao, Jiao J bo, et al. A novel Aptamer-induced CHA amplification strategy for ultrasensitive detection of Staphylococcus aureus and NIR-triggered photothermal bactericidal Activity based on aptamer-modified magnetic Fe3O4@AuNRs. Sensors and Actuators B: Chemical. 2023 May;382:133554.

5. Abbaspour A, Norouz-Sarvestani F, Noori A, Soltani N. Aptamer-conjugated silver nanoparticles for electrochemical dual-aptamer-based sandwich detection of staphylococcus aureus. Biosensors and Bioelectronics. 2015 Jun;68:149–55.

6. Marin M, Rizzotto F, Léguillier V, Péchoux C, Borezee-Durant E, Vidic J. Naked-eye detection of Staphylococcus aureus in powdered milk and infant formula using gold nanoparticles. Journal of Microbiological Methods. 2022 Oct;201:106578.

7. Zhao W, Yang S, Zhang D, Zhou T, Huang J, Gao M, et al. Ultrasensitive dual-enhanced sandwich strategy for simultaneous detection of Escherichia coli and Staphylococcus aureus based on optimized aptamers-functionalized magnetic capture probes and graphene oxide-Au nanostars SERS tags. Journal of Colloid and Interface Science. 2023 Mar;634:651–63.

8. Kim YS, Song MY, Jurng J, Kim BC. Isolation and characterization of DNA aptamers against Escherichia coli using a bacterial cell–systematic evolution of ligands by exponential enrichment approach. Analytical Biochemistry. 2013 May;436(1):22–8.

9. Huang Y, Chen X, Xia Y, Wu S, Duan N, Ma X, et al. Selection, identification and application of a DNA aptamer against Staphylococcus aureus enterotoxin A. Anal Methods. 2014;6(3):690–7.

10. DeGrasse JA. A Single-Stranded DNA Aptamer That Selectively Binds to Staphylococcus aureus Enterotoxin B. Kourentzi K, editor. PLoS ONE. 2012 Mar 16;7(3):e33410.

11. Mousavi Nodoushan S, Nasirizadeh N, Amani J, Halabian R, Imani Fooladi AA. An electrochemical aptasensor for staphylococcal enterotoxin B detection based on reduced graphene oxide and gold nano-urchins. Biosensors and Bioelectronics. 2019 Feb;127:221–8.

12. Han SR, Lee SW. In vitro selection of RNA aptamer specific to Staphylococcus aureus. Ann Microbiol. 2014 Jun;64(2):883–5.

13. Alizadeh N, Memar MY, Mehramuz B, Abibiglou SS, Hemmati F, Samadi Kafil H. Current advances in aptamer-assisted technologies for detecting bacterial and fungal toxins. J Appl Microbiol. 2018 Mar;124(3):644–51.

14. Chen J, Huang Z, Luo Z, Yu Q, Xu Y, Wang X, et al. Multichannel-Structured Three-Dimensional Chip for Highly Sensitive Pathogenic Bacteria Detection Based on Fast DNA-Programmed Signal Polymerization. Anal Chem. 2018 Oct 16;90(20):12019–26.

15. Ferreira IM, De Souza Lacerda CM, De Faria LS, Corrêa CR, De Andrade ASR. Selection of Peptidoglycan-Specific Aptamers for Bacterial Cells Identification. Appl Biochem Biotechnol. 2014 Dec;174(7):2548–56.

16. Bakhshandeh F, Saha S, Sen P, Sakib S, MacLachlan R, Kanji F, et al. A universal bacterial sensor created by integrating a light modulating aptamer complex with photoelectrochemical signal readout. Biosensors and Bioelectronics. 2023 Sep;235:115359.

17. Moon, J.; Kim, G.; Park, S.B.; Lim, J.; Mo, C. Comparison of Whole-Cell SELEX Methods for the Identification of *Staphylococcus Aureus*-Specific DNA Aptamers. *Sensors* **2015**, *15*, 8884–8897. https://doi.org/10.3390/s150408884

18. Chen, C.; Li, M.; Xing, Y.; Li, Y.; Joedecke, C.C.; Jin, J.; Yang, Z.; Liu, D. Study of pH-Induced Folding and Unfolding Kinetics of the DNA i-Motif by Stopped-Flow Circular Dichroism. *Langmuir* **2012**, *28*, 17743–17748

19. Zhang Y, Liu Y, Yang Y, Li L, Tao X, Song E. Rapid detection of pathogenic bacteria based on a universal dual-recognition FRET sensing system constructed with aptamer-quantum dots and lectin-gold nanoparticles. Chinese Chemical Letters. 2023 Aug;34(8):108102.

20. Shin HS, Gedi V, Kim JK, Lee D ki. Detection of Gram-negative bacterial outer membrane vesicles using DNA aptamers. Sci Rep. 2019 Sep 11;9(1):13167.

21. Niles JC, Marletta MA. Utilizing RNA Aptamers To Probe a Physiologically Important Heme-Regulated Cellular Network. ACS Chem Biol. 2006 Sep 1;1(8):515–24.

22. Bruno JG, Carrillo MP, Phillips T, Andrews CJ. A Novel Screening Method for Competitive FRET-Aptamers Applied to E. coli Assay Development. J Fluoresc. 2010 Nov;20(6):1211–23.

23. Queirós RB, de-los-Santos-Álvarez N, Noronha JP, Sales MGF. A label-free DNA aptamer-based impedance biosensor for the detection of E. coli outer membrane proteins. Sensors and Actuators B: Chemical. 2013 May;181:766–72.

24. Jiang D, Yang C, Fan Y, Polly Leung HM, Inthavong K, Zhang Y, et al. Ultra-sensitive photoelectrochemical aptamer biosensor for detecting E. coli O157:H7 based on nonmetallic plasmonic two-dimensional hydrated defective tungsten oxide nanosheets coupling with nitrogen-doped graphene quantum dots (dWO3•H2O@N-GQDs). Biosensors and Bioelectronics. 2021 Jul;183:113214.

25. Kaur H, Shorie M, Sabherwal P. Biolayer interferometry-SELEX for Shiga toxin antigenic-peptide aptamers & detection via chitosan-WSe2 aptasensor. Biosensors and Bioelectronics. 2020 Nov;167:112498.

26. Kim YS, Chung J, Song MY, Jurng J, Kim BC. Aptamer cocktails: Enhancement of sensing signals compared to single use of aptamers for detection of bacteria. Biosensors and Bioelectronics. 2014 Apr;54:195–8.

27. Schmitz FRW, Cesca K, Valério A, De Oliveira D, Hotza D. Colorimetric detection of Pseudomonas aeruginosa by aptamer-functionalized gold nanoparticles. Appl Microbiol Biotechnol. 2023 Jan;107(1):71–80.

28. Zheng H, Sheng R, Li H, Ahmad W, Chen Q. Rapid and selective detection of Bacillus cereus in food using cDNA-based up-conversion fluorescence spectrum copy and aptamer modified magnetic separation. Spectrochimica Acta Part A: Molecular and Biomolecular Spectroscopy. 2022 Feb;267:120618.

29. Duan N, Ye M, Lu M, Chen X, Wu S. DNA aptamers selection and characterization for development of impedimetric aptasensor for Bacillus cereus at different growing stages. Advanced Agrochem. 2023 Sep;2(3):284–90.

30. Zhou Z, Lan X, Zhu L, Zhang Y, Chen K, Zhang W, et al. Portable dual-aptamer microfluidic chip biosensor for Bacillus cereus based on aptamer tailoring and dumbbell-shaped probes. Journal of Hazardous Materials. 2023 Mar;445:130545.

31. Effah CY, Ding L, Tan L, He S, Li X, Yuan H, et al. A SERS bioassay based on vancomycin-modified PEI-interlayered nanocomposite and aptamer-functionalized SERS tags for synchronous detection of Acinetobacter baumannii and Klebsiella pneumoniae. Food Chemistry. 2023 Oct;423:136242.

32. Abedi R, Raoof JB, Mohseni M, Bagheri Hashkavayi A. Development of a label-free impedimetric aptasensor for the detection of Acinetobacter baumannii bacteria. Analytical Biochemistry. 2023 Oct;679:115288.

33. Vishwakarma A, Meganathan Y, Ramya M. Aptamer-based assay for rapid detection, surveillance, and screening of pathogenic Leptospira in water samples. Sci Rep. 2023 Aug 17;13(1):13379.

34. Rizzotto F, Marin M, Péchoux C, Auger S, Vidic J. Colorimetric aptasensor for detection of Bacillus cytotoxicus spores in milk and ready-to-use food. Heliyon. 2023 Jul;9(7):e17562.

35. Frohnmeyer E, Frisch F, Falke S, Betzel C, Fischer M. Highly affine and selective aptamers against cholera toxin as capture elements in magnetic bead-based sandwich ELAA. Journal of Biotechnology. 2018 Mar;269:35–42.

36. Luo P, Liu Y, Xia Y, Xu H, Xie G. Aptamer biosensor for sensitive detection of toxin A of Clostridium difficile using gold nanoparticles synthesized by Bacillus stearothermophilus. Biosensors and Bioelectronics. 2014 Apr;54:217–21.

37. Hu J, Shen Z, Tan L, Yuan J, Gan N. Electrochemical aptasensor for simultaneous detection of foodborne pathogens based on a double stirring bars-assisted signal amplification strategy. Sensors and Actuators B: Chemical. 2021 Oct;345:130337.

38. Wang W, Yuan W, Wang D, Mai X, Wang D, Zhu Y, et al. Dual-mode sensor based on the synergy of magnetic separation and functionalized probes for the ultrasensitive detection of *Clostridium perfringens*. RSC Adv. 2022;12(39):25744–52.
